# Presence of Perfluoroalkyl and Polyfluoroalkyl Substances (PFAS) in Food Contact Materials (FCM) and Its Migration to Food

**DOI:** 10.3390/foods10071443

**Published:** 2021-06-22

**Authors:** Arabela Ramírez Carnero, Antía Lestido-Cardama, Patricia Vazquez Loureiro, Letricia Barbosa-Pereira, Ana Rodríguez Bernaldo de Quirós, Raquel Sendón

**Affiliations:** Department of Analytical Chemistry, Nutrition and Food Science, Faculty of Pharmacy, University of Santiago de Compostela, 15782 Santiago de Compostela, Spain; arabella.r.c@gmail.com (A.R.C.); antia.lestido@usc.es (A.L.-C.); patriciavazquez.loureiro@usc.es (P.V.L.); letricia.barbosa.pereira@usc.es (L.B.-P.); ana.rodriguez.bernaldo@usc.es (A.R.B.d.Q.)

**Keywords:** perfluoroalkyl substances, polyfluoroalkyl substances, perfluorooctanoic acid, perfluorosulfonic acid, food packaging, PTFAS migration

## Abstract

Perfluoroalkyl and polyfluoroalkyl substances (PFAS) are synthetic chemical compounds widely used in different industry fields including food contact materials (FCM), providing resistance to fat and humidity, and non-stick properties. PFAS enter into the food chain directly from the intake of contaminated food or indirectly from the migration of the FCM into the food. This exposure published in different research highlights a public health concern. Therefore, it is necessary to perform analysis of the content of different FCM and evaluate the migration from the FCM under normal conditions of use and storage. This bibliographical review proves that different perfluoroalkyl and polyfluoroalkyl compounds are detected in fast food packaging, microwave popcorn bags, and frying pans, among others. Furthermore, it shows the conditions or factors that favor the migration of the PFAS from the FCM into the food.

## 1. Introduction

The perfluoroalkylated and polyfluoroalkylated substances (PFAS) are a series of synthetic chemical compounds formed by a hydrophobic alkyl chain of variable size (usually between 4 and 16 carbons), which can be partially or totally fluorinated, i.e., they contain one or more atoms of carbon in which the hydrogen atoms have been replaced by fluorine atoms and a final hydrophilic group [1].

In the case that the hydrophobic chain is partially fluorinated, they are called polyfluoroalkyl substances, while if the chain is totally fluorinated, except in the H atoms whose substitution would modify the nature of a functional group, they are called perfluoroalkyl substances. Polyfluoroalkyl substances can transform, under appropriate conditions, into perfluoroalkyl substances such as by degradation in the environment [2].

There are more than 4500 manufactured substances that meet the definition of PFAS, but only a few of these synthetic substances have been the object of study and legislation by the environmental and health authorities of different countries since both PFAS and their impurities are released into the environment in the production, use, and disposal processes. They are considered environmental pollutants and have been associated with different types of cancer, developmental toxicity, and immunotoxicity. For example, there are epidemiological studies linking exposure to long-chain perfluorooctanoic acid (PFOA) and/or perfluorooctane sulfonic acid (PFOS) with kidney and testicular cancer, low birth weight, thyroid disease, and immunotoxicity in children [3].

Most PFAS are acids that can be in their protonic or anionic form or even as a mixture of both. In the review of the terminology proposed by different authors, it was established to name all PFAS as acids instead of carboxylates, sulfonates, etc. [2].

**Figure 1:**
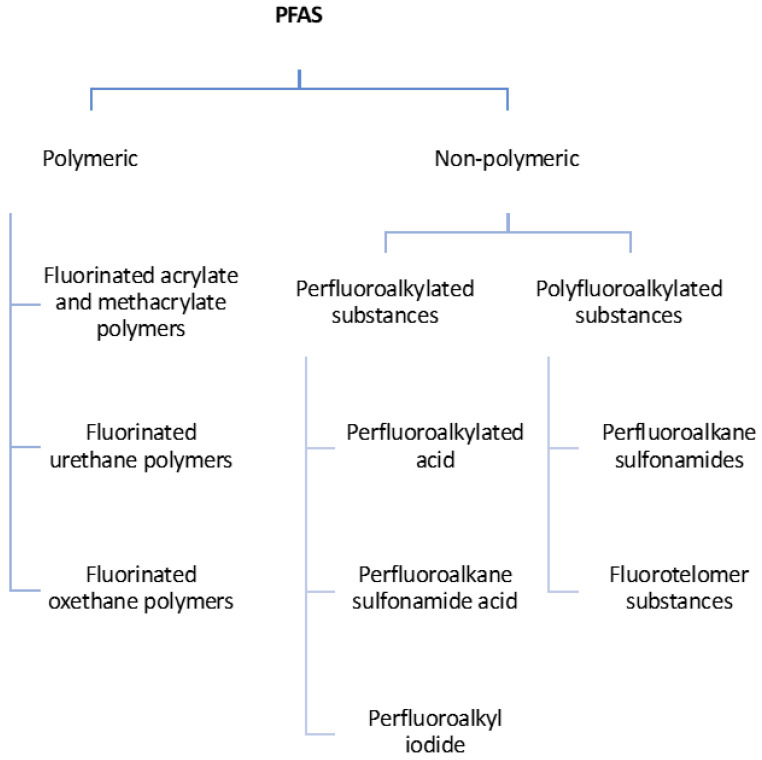
Classification of perfluoroalkylated and polyfluoroalkylated substances [2].

Moreover, PFAS can be divided into two large groups: non-polymeric and polymeric PFAS, as shown in Figure 1.

**Figure 2:**
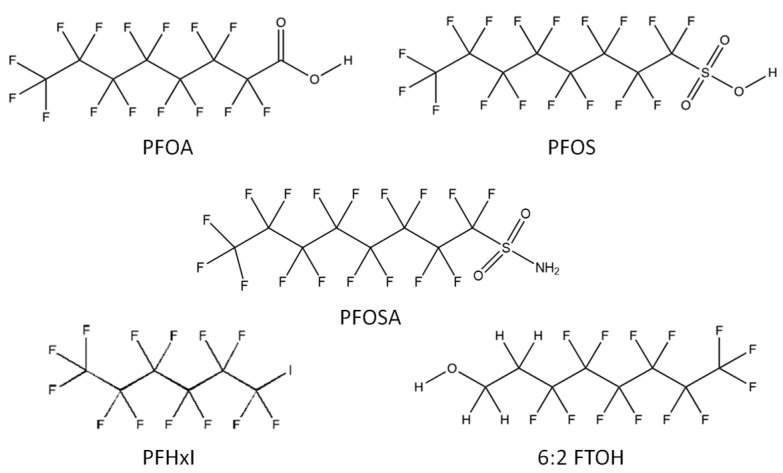
Chemical structure of some PFAS.

1. Non-polymeric perfluoroalkylated substances:
  1.1. Perfluoroalkylated acids (PFAA) most representatives:
    1.1.1. Perfluoroalkyl carboxylic acids (PFCAs), also known as perfluorocarboxylic acids or perfluoroalkanoic acids, have the general chemical formula C_n_F_2n+1_COOH. Some PFCAs are products of the abiotic or biotic degradation of certain PFAS. Perfluoroctanoic acid (PFOA) is the most frequently discussed compound (Figure 2). The ammonium salt, ammonium perfluorooctane (APFO), has been used in the production of fluoropolymers such as polytetrafluoroethylene (PTFE), commonly known as Teflon^®^, which is used in kitchen utensils such as frying pans and pots due to its low coefficient of friction and its impermeability. Other acids are perfluorononanoic acid (PFNA), manufactured primarily for use as a fluorinated surfactant; perfluoroundecanoic acid (PFUnDA) and perfluorotridecanoic acid (PFTrDA) [2].
    1.1.2. Some perfluoroalkane sulfonic acids (PFSA) are perfluorobutane sulfonic acid (PFBS) and perfluorooctane sulfonic acid (PFOS) (Figure 2). PFOS has been the most studied since it has been found in biota worldwide. Besides, both PFOS and its salts have been classified as persistent, bioaccumulative, and toxic substances. Furthermore, it has been added to Annex B of the list of persistent organic contaminants of the Stockholm Convention, therefore its production and use is restricted [4].
    1.1.3. Perfluoroalkyl phosphonic and phosphinic acids (PFPAs and PFPIAs) are surfactants manufactured for a range of consumer and industrial uses. PFPAs have been widely detected in environmental waters.
  1.2. Perfluoroalkane sulfonamide (PFASAs) such as perfluorooctane sulfonamide (PFOSA) is the major raw material for surfactant and surface protection products
  1.3. Perfluoroalkyl iodide (PFAIs): It is a family of fluorotelomers whose degradation is a potential source of PFCA. One example is perfluorohexyl iodide (PFHxI), which chemical structure is C_6_F_13_I.

2. Non-polymeric polyfluoroalkylated substances:
  2.1. Perfluoroalkane sulfonamides, such as N-methylperfluorooctane sulfonamide (N-MeFOSA), N-ethyl perfluorobutane sulfonamide (EtFBSA), and N-butyl perflorooctane sulfonamide (BuFOSA).
  2.2. Fluorotelomer substances:
    2.2.1. Fluorotelomer alcohols of different chain lengths (FTOH) provide water and oil repellency and are used in materials in contact with food as non-stick agents. For example, 1H,1H,2H,2H-perfluorooctan-1-ol (6:2 FTOH), 1H,1H,2H,2H-perfluoro-decan-1-ol (8:2 FTOH) and 1H,1H,2H,2H-perfluoro-1-dodecanol (10:2 FTOH). In the “*X:Y*” designation used for naming fluorotelomer substances, *X* is the number of perfluorinated C atoms, while *Y* is the number of non-fluorinated C atoms that originate from the commercial synthesis.
    2.2.2. Polyfluoroalkyl phosphoric acid esters, polyfluoroalkyl phosphates, and fluorotelomer phosphates (PAP) may be referred as polyalkyl phosphoric acid monoesters or fluorotelomer monophosphates (monoPAPs), and polyalkyl phosphoric acid diesters or fluorotelomer diphosphates (diPAPs). These compounds have been used as waterproofing agents for food contact paper [2]. Their approval as defoaming adjuvant in pesticide formulations has been rescinded [5].

The carbon and fluorine bonds of these substances make them chemically stable, extremely resistant to degradation even at high temperatures, and resistant to water and oil. Therefore, perfluoroalkylated substances are widely used both in industry and in materials in contact with food, such as non-stick kitchen utensils and lipophobic and hydrophobic coatings for paper and cardboard products. In addition, they are used in anti-stain coatings, waterproof fabrics, furniture paints and varnishes, fire extinguishing foams, and floor polishes due to their hydrophobic and lipophobic nature. High levels of PFAS, most commonly PFOS, PFHxA, PFOA, and PFDoA, have been found in ski waxes, leather samples, and carpets [6]. They are mainly used as raw materials for surfactants and surface protection products.

PFAS bioaccumulate and pass the food chain through ingestion of contaminated food and beverages being the main route of direct exposure to PFAS. There is also an indirect exposure from consumption of foods that have been in contact with materials that had PFAS [7,8].

European Food Safety Authority (EFSA) collected monitoring data during the years 2000–2009 and in 2011 published a report on the presence of PFAS in 4881 samples collected in seven EU Member States. In this report, it was revealed that the highest mean contamination of PFOS (216 μg kg^−1^), PFNA (10.3 μg kg^−1^), PFOA (7.1 μg kg^−1^), PFDA (6.0 μg kg^−1^), and PFDoDA (3.7 μg kg^−1^) was found in remains of game animals, mainly in the liver of wild pig. PFOS and PFOA were found sporadically in farm animals and poultry. In fish, PFOS and PFOSA were also found, with mean values of 47 and 15 μg kg^−1^, respectively. The concentrations of PFAS in farmed fish were lower than in wild fish, this could be due to contamination of rivers and seas by the industry that produces these substances. Shellfish are considered an important source of direct consumption of PFAS since there is a high frequency of positive results among them, although the number of samples analyzed is small [9]. Studies reveal that production of paper products may be a major PFAS point source, being an important vector for PFAS in the aquatic environment [10].

At the same time the European Commission published the Recommendation 2010/161/EU in which Member States must monitor the presence of perfluoroalkylated substances in food during 2010 and 2011. This surveillance had to include a wide variety of foods that reflected the eating habits of each region.

Later, and due to their high persistence in the environment and the global distribution of these substances, the EFSA Technical Panel on Contaminants in the Food Chain (CONTAM) recommended collecting data on the presence of PFAS in different food products and the human body, especially evaluating the exposition [11]. 

After ingestion and excretion, fluorinated substances degraded end up in wastewater, so they are likely to further contaminate the environment and re-enter the food chain.

Since the most frequent PFAS found in food samples are PFOS and PFOA, CONTAM established a tolerable daily intake (TDI) of 150 ng kg^−1^ body weight (bw) per day for PFOS and 1500 ng kg^−1^ bw per day for PFOA [11]. TDIs were based on a non-observed adverse effect level (NOAEL) of 0.03 mg kg^−1^ bw per day for PFOS and 0.06 mg kg^−1^ bw per day for PFOA. For the rest of PFAS found in food, there is no tolerable daily intake, and then dietary exposure studies are focused on these two substances. Chronic dietary exposure to the rest of PFAS is expected to be less than exposure to PFOA and PFOS.

In the dietary exposure report published by EFSA in 2012, it was observed that in the case of PFOS, the estimates of dietary exposure were less than 3.5% of the TDI for average consumers and less than 6.7% of the TDI for high consumers, while in the case of PFOA, the exposure represented less than 0.3% for medium consumers and less than 0.5% for large consumers. Compared with the TDI established by the CONTAM, it can be observed that for both average and large consumers, the daily intake is below, with fish, shellfish, meat, and meat products being the main contributors to this exposure [11].

In eastern countries, the exposure is higher than in western countries, mainly because they have a higher contamination of the rivers used to irrigate rice crops, the basis of their diet. Exposure to PFAS in the population of Taiwan is 0.46 ng kg^−1^ bw per day for PFOS and 85.1 ng kg^−1^ bw per day for PFOA. In this country, the TDIs are exceeded in pregnant women due to the consumption of pork liver, which is not studied in Western countries because it is not a frequent food [12].

It is important to note that migration of PFAS from FCM is not considered in the exposure calculations, possibly due to the lack of data.

The objective of this work was to carry out a bibliographic review on perfluoroalkyl and polyfluoroalkyl substances to know both their presence in FCM and their migration data into food.

## 2. Legislation

Materials that are intended to come into contact with food are regulated in the Regulation (EC) No. 1935/2004. This regulation establishes that materials and objects intended to extend the shelf life or maintain or improve the state of the food packaging must be manufactured following good manufacturing practices so that, under foreseeable conditions of use, they do not transfer their components in quantities that may represent a danger to human health, not causing the alteration of the organoleptic conditions of food or causing an unacceptable modification of its composition [13].

In 2010, PFOS was included in the Stockholm Convention, which is intended to control persistent organic pollutants to protect human health and the environment, so its use should be restricted as much as possible. In 2020, the EU published the Regulation (EU) 2020/784 that amends current EU legislation and restricts the use of persistent organic contaminants. The new regulation specifically limits the use of PFOA, its salts, and PFOA-related compounds. This regulation sets a maximum concentration of 0.025 mg kg^−1^ for PFOA and any of its salts, and a maximum concentration of 1 mg kg^−1^ for PFOA-related compounds or a combination of those compounds [14]. This was also taken into account when issuing the Commission Recommendation (EU) 2019/794, that recommends the investigation of the presence of PFAS in FCM, specifically its presence in paper and board-based materials, such as those used to wrap fast food and takeaway and bakery products, and also popcorn bags [15].

## 3. PFAS in Food Contact Materials

Food contact materials (FCM) are materials intended to come in contact with food during its transport, storage, conservation, handling, or manufacture. Food packaging is used in the food industry to protect food from chemical, physical, and microbiological deterioration, which preserves the quality, nutritional and hygienic properties of the food, and in some cases, it even facilitates the processing of the food [16].

PFAS are widely used in FCM because its carbon-fluorine bonds provide high resistance to degradation even at high temperatures. FCM that contain PFAS, such as fast-food packaging and microwave popcorn bags or non-sticks utensils, can indirectly contribute to dietary exposure through the migration into food, which can be a food safety concern [3]. Consumption of restaurant food and popcorn was associated with higher serum levels of PFAS as a consequence of PFAS migration from the FCM [17].

FCM that contain perfluoroalkylated and polyfluoroalkylated substances can represent a health risk, since these can migrate into food and be ingested, and also being a risk to the environment, since they can be released in the process of eliminating these materials [18].

PFAS were first used in the 1940s and 1950s, and since then, they have been used in paper and cardboard packaging to provide resistance to water, oil, and fat. It is also a common practice to avoid the transfer of fats from the food to paper or cardboard packaging, improving its presentation. Since 1960, PFCAs have been used also as surfactants, such as PTFE (Polytetrafluoroethylene), which is used as a non-stick coating in pans or pots [19]. PTFE prevents food from sticking during the cooking process but also makes the utensil washing process easier. 

Both PFOS and PFOA are the most studied PFCAs since they have been found in higher concentrations in water, soil, and food [20,21]. Therefore, there are several studies of its presence in FCM, and they are summarized in Tables 1 and 2. 

The safety of fluorocarbon resins, including perfluoroalkyl and polyfluoroalkyl, such as PTFE, mostly known as Teflon^®^, of pans and other kitchen utensils has been the aim of some research studies, since these materials reach very high temperatures, which promote the migration.

### Analytical Determination

PFAS, which are used in food contact paper and board, have been described to be highly persistent, bioaccumulative, and toxic. To determine the sources of PFAs exposure through paper and board, analytical methods are needed, not only to analyze them, but also their impurities and their degradation products. Consequently, new fluorinated products can appear, and there is currently no universal analysis methodology to identify all PFAs [22]. Polyfluorinated surfactants are complex mixtures that represents an analytical chance and require high performance analytical techniques to determine all the related substances that could be present in these FCM [23].

There are several non-specific methods to determine the total fluorine in samples. Spectroscopic methods can be used such as SSS (Sliding Spark Spectroscopy), NMR (Nuclear Magnetic Resonance), Raman spectroscopy, PIGE (particle-induced gamma-ray spectroscopy), or chromatographic methods such as CIC (Combustion Ion Chromatography with a conductivity detector), being PIGE and ion chromatography the most suitable quantitative methods to determine the amount of total organic fluorine in a sample [3,24]. In addition to this technique, Instrumental Neutron activation analysis (INAA) is another technique for total fluorine determination [25]

As fluorine may be present in other fluorinated compounds or as inorganic fluorine, it is necessary to determine PFAS. The identification of these types of compounds is difficult since they are present in low concentrations, and the samples usually contain a mixture of different types of PFAS [26].

The results obtained are usually expressed as analyte weight/material surface or transformed in analyte weight/food or simulant weight considering that 1 kg of food is generally in contact with 6 dm^2^ of material (or the real relationship if it is known), also as analyte weight/material weight [27].

The determination of the content in the materials is usually performed by liquid chromatography (LC or UPLC) coupled to a mass spectrometry (MS) detector. Tandem mass spectrometry is also employed [28,29,30,31].

Some laboratories in Denmark and Sweden have established liquid chromatography coupled to mass spectrometry (LC-MS) as a method to determine PFAA, some derivatives of PFOS, and total organic fluorine [22].

Liquid chromatography methods coupled to triple quadrupole mass spectrometry (LC-QqQ) and coupled to quadrupole time-of-flight mass spectrometry (LC-QTOF) have been developed, both LC/MS-MS based techniques can detect PFAS at lower levels in the packaging [32,33].

Fluorinated compounds are intentionally added to materials that are in direct contact with food. Paper samples were analyzed by Shaider et al. (2017) and total fluorine was not detected in samples that were not intended for food contact, while in those designed for food contact, fluorine was found to be higher than 16 nmol cm^−2^ [3].

Microwave popcorn packaging has been highly studied since they are subjected to high temperatures during their preparation and are used into contact with fatty acids. In the study of Monge Brenes et al. (2019) several works that study the presence of the most monitored perfluoroalkylated substances, both PFAS and PFOS, in this type of packaging between 2005 and 2018 are included and further commented below. The results of this study suggest a reduction in the concentration of PFOS and PFOA in microwave packaging [34].

Dolman et al. (2011) detected PFOA in a popcorn bag, as well as other PFCs such as PFHxA, PFHpA, PFNA, PFDA, and PFUA, showing that popcorn bags were treated with PFC to increase their resistance to temperature and fats. In this case, after using the microwave, no PFCs were detected in the popcorn, suggesting that the PFCs remained in the bag and therefore did not migrate to the popcorn [35].

Granby et al. (2018) carried out a similar study in the Technical University of Denmark, in which three samples of popcorn bags were analyzed. Among the 35 samples analyzed, including baking dishes, fast-food packaging, popcorn bags, and muffin packaging, the presence of different types of PFAS was detected in 13 samples. The sum of the different PFCAs (PFPeA, PFHxA, PFHpA, PFOA, PFNA, PFDA, PFUnDA, PFDoDA, PFTeA, PFTrA) detected in 10 samples ranged between 0.01 and 13.1 μg kg^−1^ of food. Fluorotelomer alcohols (FTOH) were detected in nine samples at concentrations of 0.8–13.5 μg kg^−1^ of food, 6:2 FTOH being the most frequently detected as well as the one with higher concentration quantified in each sample. The highest concentrations of PFAS (PFCA and FTOH) were detected in two muffin packaging with values of 6.1 and 14.1 μg kg^−1^, respectively, and in three baking dishes with values of 19.4, 13.3, and 25.3 μg kg^−1^, respectively. Migration tests were also carried out on these five samples [29].

The standards employed are not the same in western countries as in eastern countries. In American and European countries, shorter-chain PFCAs, PFBA, PFPeA, PFHxA are used, while in Asian countries, especially in China, longer chain PFCAs, PFOA, PFNA, PFDA are used [36].

Yuan et al. (2016) analyzed 42 samples of food packaging made of paper and aluminum, including fast-food wrappers, popcorn packaging, pizza and sandwich boxes, baking paper and aluminum wrappers. They used pressurized liquid extraction (PLE) followed by LC-MS/MS and the isotope dilution method to quantify determine 12 PFCs: PFBA, PFPeA, PFHxA, PFHpA, PFOA, PFNA, PFDA, PFUnDA, PFDoA, PFBS, PFHxS, and PFOS and 5 PFCs: PFTrDA, PFTeDA, PFHxDA, PFoDA, and PFD. PFHxA was detected in a cup of ice cream, PFBA, PFHxA, PFHpA, PFNA, PFDA, and PFDoA were detected in fast-food wrappers, while the highest values were obtained in bags of microwave popcorn. In this case, neither PFOA nor PFOS were significantly detected, unlike those that occurred in the European studies mentioned before [28].

The same researchers previously mentioned compared the presence of FTOH; both 6:2 FTOH, 8:2 FTOH and 10:2 FTOH, 12:2 FTOH, 14:2 FTOH, 16:2 FTOH, and 18:2 FTOH were found in eco-friendly paper tableware, microwave popcorn bags, and other FCM such as packaging for cupcakes, cups, and cardboard boxes. It was also observed that the mean concentration of the total FTOH was higher in ecological paper tableware (2990 μg kg^−1^) than in other FCM (<0.55–38.7 μg kg^−1^) but lower than those present in the microwave paper (18,200 μg kg^−1^) [12]. 

No scientific research is available on possible PFAS contamination of paper and board FCMs when contaminated process water is used in the manufacture of paper since PFAS are ubiquitous in the environment. Some studies that determine PFAS in FCM are shown in Table 1.

**Table 1:** Determination of PFAS content in FCM.

Country	Compound	Type of Sample	Origin of Sample	Method of Determination	Concentration	Reference
Australia	PFOA PFHxA PFHpA PFNA PFDA PFUA	Popcorn bags, baking paper, box of chips, sandwich wrap, hamburger box	Local shops and fast-food restaurants	LC-MS	PFOA: 9.1 × 10^6^ g kg^−1^	[35]
EEUU	PFOA	PTFE cookware, popcorn bags, PTFE film/sealant tape.	Not specified	LC-MS LC-MS/MS	4–290 μg kg^−1^ PTFE film/sealant tape: 1800 μg kg^−1^	[37]
EEUU	PFOA PFOS	Bags of popcorn, sandwich bags	Not specified	UHPLC-QTOF	PFOS < LOD PFOA: 22.1 and 12.9 ng dm^−2^	[34]
Denmark	PFCA PFSA PAPs FTOH	Baking paper, fast-food packaging, boxes of pizza, popcorn bags, etc.	Distributors and retailers in Norway	LC-MS/MS	FTOH: 1.3 μg kg^−1^ of food	[29]
Greece	PFBA PFPeA PFHxA PFHpA PFOA PFNA PFDA PFUnDA PFDoA PFTrDA PFTeDA PFHxDA PFODA PFBS PFHxS PFOS PFDS	Fast-food packaging, sandwiches, cups, ice cream containers, baking paper, popcorn bags, etc.	Athens market and fast-food restaurants in Greece.	PLE, LC-MS/MS	PFBA: 275.84 μg kg^−1^ PFHxA: 341.21 μg kg^−1^ PFHpA: 5.19 μg kg^−1^	[38]
China	6:2 FTOH 8:2FTOH 10.2 FTOH 12:2 FTOH 14:2 FTOH 16:2 FTOH 18:2 FTOH	Bags of popcorn, materials labelled as ecological, cupcake packaging, etc.	Beijing retail market and online	UPLC-MS/MS UPLC-QTOF	Average concentration of total FTOH: 2990 μg kg^−1^	[28]

## 4. PFAS Migration into Food

FCMs can transfer substances to food through a process known as migration. Migration is a phenomenon that cannot be avoided, and it depends on many factors that obey Fick’s laws of diffusion. This migration depends on the facility of PFAS to be released by the material, on the food contact conditions such as temperature and exposure time, on the properties of the material in contact with food such as thickness, initial concentration, and diffusion coefficient, and on the interaction between the material and the compound, expressed as the coefficient of distribution between the material and the food [22,39].

Furthermore, the extent of migration to food depends on the concentration, mass fractions, type, and length of the PFAS chain, as well as the nature of food while the migration increases with the use of high temperatures and the use of fats even if the contact time between the material and the food is short [3,40].

The ingestion of this type of substances is a food safety problem, so it is necessary to evaluate the migration of PFAS from FCM into the food in usual conditions of cooking and storage. The first step is the release of PFAS from the surface of the material, which depends on whether PFAS is absorbed into the material or on the material surface. For the release of these substances, the surface should be wet and break the bonds between the substance and the surface [22,28]. The second step for the migration of PFAS is the dissolution of the compounds into food.

The determination of PFAS in food is difficult because the food can have this type of chemical substances before its packaging or processing, since they are environmental pollutants. To carry out these migration tests, it is advisable to use food simulants since they are simpler analytical matrices than food [41].

Furthermore, it is necessary to ensure that there is no contamination from the material laboratory used. Some studies replace, for example, the PTFE filter used at the entry of the mobile phase in LC-MS with a paper filter free of Teflon [30].

Migration tests were carried out under the following conditions: for example, in the case of a dish which contains food to be heated in a microwave, the migration conditions used were 70 °C for 2 h, since it is expected to reach high temperatures during food preparation [29]. In the case of samples such as baking paper that cannot contain liquids, a part of the sample is embedded in the lid of a stainless-steel cylinder, in which a simulant such as Tenax^®^ is used for solid foods and seal before introducing them in the oven at a suitable temperature and time [30]. For samples such as muffin wrappers, pizza boxes, and cups for hot drinks, square cuts of them are made before adding the simulant and placing them at the appropriate temperature for the time necessary for migration to occur [29], as established by the Commission Regulation (EU) No 10/2011 [16].

### 4.1. PFAS Migration Levels and Food

#### 4.1.1. Fat Content

PFAS are mostly found in foods rich in proteins, such as liver, game meat, farm animals, and fish because of the easier union with this essential nutrient [9]. However, in the migration tests, the fat content affects more than the protein content since these substances are used to increase the resistance of the material to fats, being widely used in fast-food packaging and ultra-processed foods.

Begley et al. (2005) used a PTFE sealant film with a high content of PFOA as surrogate to compare the migration after 2h in water at 100 °C and Myglyol at 100 and 175 °C. In the migration test at 100 °C higher levels of PFOA were determined (150 ng dm^−2^ vs. 120 ng dm^−2^), but in the case of Myglyol at 175 °C the quantity of PFOA was higher than in the two previous cases (710 ng dm^−2^) [37]. 

Choi et al. (2018) analyzed a total of 312 samples, including pans, bakeware, electric rice cookers, grills, and baking papers, showing that PFAS do not migrate equally to all types of food. In this case, the analytes that migrated in the highest proportion were PFODA with a concentration of 3.05 μg L^−1^ in the n-heptane simulant and PFNA with a concentration of 2.12 μg L^−1^ in 50% ethanol, simulant in which these compounds are most frequently detected. These results indicate that the migration of PFCs is more likely in alcoholic beverages or into fatty foods [30].

Elizalde et al. (2018) have studied the migration of perfluorinated compounds from paper bags to Tenax^®^ and lyophilized milk and concluded that migration of PFHxA, PFHpA, PFOA, PFNA, PFDA, PFTrDA, and PFTeDA was higher in whole milk than in low-fat milk. Both kinds of milk were freeze-dried, and low-fat milk contained 50% less fat than whole milk [31].

In the case of emulsified foods, higher migration levels are shown compared to non-emulsified fatty foods. The migration of PFAS in emulsified foods studied by Begley et al. (2008) showed that it was up to 50 times higher in emulsified foods, such as butter, compared to the migration in non-emulsified fats, such as oil. This effect was the same in the migration using food simulants, where increasing the percentage of ethanol with respect to water, from 10% to 30%, implies an increase in the migration of PFAS at 100 °C [42].

#### 4.1.2. Moisture Content

The degree of moisture of food also affects the migration of PFAS to food. Fengler et al. (2011) compared the migration in two samples of muffin masses, one of them was drier than the other. It was observed that in the case of the sample with higher moisture content, the FTOH values were lower than in the case of the muffin with lower moisture content. The authors suggest that this difference is because the FTOH evaporates, thus reducing its concentration in the food [43].

#### 4.1.3. pH

The pH of food also influences the migration of PFAS. AbulFadl et al. (2019) have studied the migration of PFOS and PFOA from utensils coated with Teflon to acidic foods, such as tomato sauce, and basic foods, such as white beans. The result showed that the amount of PFOS and PFOA that migrated was higher in tomato sauce, 18.30 μg kg^−1^ and 16.55 μg kg^−1^, respectively, while in white beans, the amount was lower, 18.08 μg kg^−1^ and 16.03 μg kg^−1^. The migration was higher to the acidic foods than to those with higher pH [44].

#### 4.1.4. Salt Content

According to the WHO, between 10 and 12 g of salt is consumed per day. This salt comes from both precooked foods and the salt added during cooking and processing. Therefore, it is of special interest to know the influence of the salt content on the migration of perfluoroalkylated substances [45].

Some authors have observed that the presence of sodium chloride increases the transfer of PFAS. Comparing the transfer of PFOS and PFOA from a non-stick utensil to food with and without salt, it was observed that the migration was higher in foods to which salt had been added (PFOS 18.08 μg kg^−1^ and PFOA 16.03 μg kg^−1^) than in which no salt was added (PFOS 13.11 μg kg^−1^ and PFOA 9.28 μg kg^−1^) [44].

### 4.2. PFAS Migration Levels and PFAS Chemistry

Some studies demonstrate that the length of the fluorinated chain can influence the migration of different compounds. 

The migration of both PFCA and FTOH of various chain lengths from paper and cardboard to food simulants of different solutions of ethanol:water (10:90, 30:70 and 50:50) was studied. For example, the migration of 6:2 FTOH to 10% ethanol was 0.00024 μg cm^−2^ and at 50% ethanol was 0.013 μg cm^−2^, increasing with the proportion of ethanol. It was also observed that the migration from paper and cardboard to water and different ethanol solutions was higher when the fluorinated chain is smaller in comparison with the migration of its long-chain analogues, for example, the migration of 6:2 FTOH to the simulant 30% ethanol was 0.00279 μg cm^−2^, while for 16:2 FTOH was 9 × 10^−6^ μg cm^−2^ [19,28]. 

### 4.3. PFAS Migration Levels and Food Contact Conditions

#### 4.3.1. Time and Temperature

The contact time between the FCM and the food is important to determine the migration of the PFAS since this time is short in fast-food or take away food packaging and can be longer if the material is used to cook or process food.

Furthermore, the water at room temperature has a high surface tension, so it is not able to wet the paper and/or the cardboard and releasing fluorinated substances, while at a high temperature its surface tension decreases and favors the release of chemicals from the surface. In this way, PFAS are released by hydrolysis, which is accelerated by heating.

In the study carried out by Granby et al. (2018) at the Technical University of Denmark, 35 samples were analyzed, as commented before. Migration test was carried out with the five samples that had a higher concentration of PFAS in two muffin packaging, with values of 6.1 and 14.1 μg kg^−1^, and in three baking dishes from the same manufacturer from the United States, where the highest concentrations of PFAS were found: 19.4, 13.3, and 25.3 μg kg^−1^. The migration test was performed in triplicate for 2 h at 70 °C in 50% ethanol:water [29]. The results showed higher values of the migrated PFAS than the results obtained in the previous analysis of the materials. In the case of one muffin packaging, 5.3 μg of FTOH kg^−1^ of food was obtained in the sample, while in the migration test was 14.4 μg kg^−1^ food. The authors justify this fact due to the higher temperature used in the migration test, 70 °C, than that used in the extraction, which was 60 °C [29].

When the migration of PFOA at 100 °C and at 175 °C to Miglyol is compared, using a surrogate film with a high amount of PTFA, results showed that the migration at 175 °C was seven times higher than at 100 °C (Begley et al., 2005). In another study performed with butter, PAP was not detected in the migration test at 5 °C, though PAP was detected in butter in the 20 °C test [42].

The migration of different compounds to the simulant Tenax^®^ was compared, at different temperatures, by Elizalde et al. (2018). The authors observed that after migration at 160 °C, the migration doubled, reaching up to 25% of the initial amount observed in the paper [31].

In the muffin packages, the values of each FTOH, 6:2 FTOH, 8:2 FTOH, and 10:2 FTOH were higher after the migration test than the initial values of the FTOH found initially, which means that production occurs in FTOH from producing compounds with increasing temperature [43].

Yuan et al. (2016) have also studied the migration from paper bowls to different food simulants, including water, solutions of ethanol:water in different proportions (10:90, 30:70, and 50:50 *v*/*v*) and oil. The food material was preheated to 100 °C, then 10 mL of the simulants were added and kept at room temperature for 15 min, thus recreating the usual conditions of use [28].

#### 4.3.2. Repeated Use 

Fast-food packaging, popcorn bags, muffin wrappers, and baking paper are usually disposable, so the migration is evaluated after a single use. This does not happen with the non-stick articles such as pans, pots, and oven racks, since these utensils are used repeatedly over a long period of time.

The migration of PFSO and PFOA to food from non-stick cookware repeatedly used was analyzed. In one of the studies shown in Table 2, it was observed that the concentration of analytes increases with the increasing number of exposures. In the first use, the concentration of PFSO in an acidic food with salt was 18.30 μg kg^−1^ and for PFOA 16.55 μg kg^−1^, and after being used five times, the concentrations found for PFSO and PFOA were 39.55 μg kg^−1^ and 34.52 μg kg^−1^, respectively, and after being used ten times 60.33 μg kg^−1^ and 54.21 μg kg^−1^ [44].

**Table 2:** Migration levels of PFAS found in foods or food simulants.

Country	Compound	Type of Samples	Origin of Samples	Method of Determination	Food/Food Simulant	Results	Reference
Korea	PFPeA PFBS PFHxA PFHpA PFHxS PFOA PFNA PFOS PFDA PFUnDA PFDS PFDoDA PFTrDA PFTeDA PFHxDA PFODA	Frying pans, baking utensils grills, baking paper	Large and local markets	LC-MS/MS	N-heptane, 50% ethanol, water and 4% acetic	PFODA: 3.05 μg L^−1^ in n-heptane PFNA: 2.12 μg/L in 50% ethanol	[30]
Spain	PFBA PFPeA L-PFBS PFHxA PFHpA L-PFHxS PFOA L-PFHpS PFNA L-PFOS PFDA PFUnDA L-PFDS PFDoDA PFTrDA PFTeDA	Paper bags	Local markets	UPLC-MS/MS	Tenax^®^ Whole milk and low-fat milk	Increased migration in whole milk. Migration to Tenax^®^ was 8% at 80 °C, 10% at 120 °C and 25% at 160 °C	[31]
EEUU	PFOA Flurotelomers	Popcorn bags	Not specified	LC/MS LC-MS/MS	Popcorn oil, Myglyol	Migration levels of fluortelomers of 1.4 mg kg^−1^ before and 2.1 mg kg^−1^ after heating	[37]
EEUU	PFOS	Non-stick paper	EEUU retail markets	LC/MS	Ethanol/water (10, 20, 25 and 30% ethanol)	Increased migration in emulsified foods	[42]
Germany	FTOH	Paper Muffins	Not specified	GC/CI-MS	Tenax Muffins	The higher the humidity the higher the FTOH migration	[43]
Egypt	PFOS PFOA	Tefal kitchen utensils	Not specified	LC-MS	Fresh tomatoes as acidic foods White beans as a staple food	Increased migration in acidic foods Increased migration in salted foods. Increased migration as the number of exhibitions increases.	[44]
China	6:2 FTOH 8:2 FTOH 10:2 FTOH 12:2 FTOH 14:2 FTOH 16:2 FTOH PFBA PFPeA PFHxA PFHpA PFOA PFNA PFDA PFUnDA PFDoDA PFTeDA PFPeDA PFHxDA PFHpDA PFODA	Popcorn bags, materials labelled as ecological, cupcake packaging, etc.	Beijing retail markets and online	UPLC-MS/MS UPLC-QTOF-MS	Water, 10%, 30% and 50% ethanol	Higher migration when the fluorine chain is smaller. Sum of 15 PFCA 0.006 μg cm^−2^ and 0.3 μg cm^−2^ for 7 FTOH	[28]
Denmark	PFPeA PFHxA PFHpA PFOA PFNA PFDA PFUnDA PFDoDA PFTeA PFTrA 6.2 FTOH 8.2 FTOH 10.2 FTOH	Dishes, Cupcake packaging	Distributors and retailers in Norway	LC-MS/MS	50% ethanol: water	Increased migration with increasing temperature	[29]

## 5. Conclusions

PFAS have been widely used, however, environmental and toxicity concerns have been raised. Moreover, some of these substances, such as PFOS and PFOA, have been restricted for use in FCM and have been replaced, mainly by other fluorinated chemical compounds.

There are some non-fluoridated alternatives to exert a barrier function on cardboard and paper, such as greaseproof vegetable parchment paper or coatings with carboxymethylcellulose (CMC), polyvinyl alcohol (PVOH), starch, aqueous dispersions of styrene-butadiene copolymers, aqueous dispersions of waxes, or hydroxyethylcellulose soluble in water (HEC) [22]. Another technique to prevent migration is to coat the material with plastic or aluminum on the side of contact with the food, as happens in multilayer milk cartons.

After this review, it can be stated that perfluoroalkylated and polyfluoroalkylated substances are still being used in FCM as fast-food wrappers, muffin packaging, baking paper, plates, and microwave popcorn bags. Some of them can reach very high temperatures and are in contact with fatty acids, which can lead in migration of PFAS into food. High levels of PFAS have been found, more frequently in PFOS, PFOA, FTOH of diverse chain lengths, PFBA, PFHxA, PFHpA, in different FCMs analyzed.

Migration depends on several factors related to the type of food in contact and increases when the fat content of the food is higher, use of emulsified foods, when the food has a low pH, and the concentration of sodium chloride is high. However, in the case of foods with high moisture content, the migration is lower. Regarding the chain length of PFAS, the migration is higher when it is shorter. The use of the FCM also influences migration, increasing with high temperatures and with the repeated use.

Microwave popcorn bags and non-stick cookware are the FCMs on which the most migration tests have been conducted and also where the highest content of PFAS were found, probably because they reach very high temperatures and are used for long periods. Moreover, the aging kitchen utensils, intended for repeated use, should be considered when evaluating the migration of PFAS. 

As the migration from FCM can contribute to dietary exposure of PFAS, it would be advisable to consider for future studies the data obtained after the implementation of the coordinated control plan proposed in the Commission Recommendation (EU) (2019/794) [15]. 

## Data Availability

Not applicable.
